# Open Heart Mitral Valve Replacement Using Transcatheter Heart Valves for Severe Mitral Annular Calcification—A Literature Review

**DOI:** 10.3390/jcdd12120491

**Published:** 2025-12-12

**Authors:** Michele D’Alonzo, Massimo Baudo, Francesco Cabrucci, Francesca Maria di Muro, Dimitrios E. Magouliotis, Beatrice Bacchi, Arian Arjomandi Rad, Andrew Xanthopoulos, Tulio Caldonazo

**Affiliations:** 1 Cardiac Surgery Unit, Poliambulanza Foundation Hospital, Via Bissolati 57, 25124 Brescia, Italy; 2 Department of Cardiac Surgery Research, Lankenau Institute for Medical Research, Main Line Health, Wynnewood, PA 19096, USA; massimo.baudo@icloud.com (M.B.); francesco.cabrucci.6@gmail.com (F.C.); magouliotisd@mlhs.org (D.E.M.); 3 Division of Cardiovascular Surgery, Peter Munk Cardiac Centre, Toronto General Hospital, University Health Network, Toronto, ON M5G 2C4, Canada; 4 Department of Medicine, Surgery and Dentistry, University of Salerno, Via Giovanni Paolo II 132, 84084 Salerno, Italy; fdimuro94@gmail.com; 5 Division of Cardiac Surgery, St. Michael’s Hospital, University of Toronto, Toronto, ON M5G 2C4, Canada; beatricebacc@gmail.com; 6 Department of Cardiothoracic Surgery, John Radcliffe Hospital, Oxford University Hospitals NHS Foundation Trust, Oxford OX3 9DU, UK; arian.arjomandirad@gmail.com; 7 Department of Cardiology, University Hospital of Larissa, 41110 Larissa, Greece; andrewvxanth@gmail.com; 8 Department of Cardiothoracic Surgery, Jena University Hospital, 07747 Jena, Germany; tulio.caldonazo@med.uni-jena.de

**Keywords:** mitral annular calcification, valve-in-MAC, transcatheter mitral valve replacement, transcatheter heart valve, paravalvular leak, LVOT obstruction

## Abstract

Mitral annular calcification makes conventional mitral valve surgery extremely challenging and has led to growing interest in less invasive alternatives such as transcatheter mitral valve replacement. Alongside percutaneous approaches, some centers have explored open transatrial implantation of transcatheter heart valves in patients with heavily calcified annuli. This systematic review examines the current evidence on this hybrid “valve-in-MAC” technique, tracing its clinical evolution, technological refinements, patient outcomes, and ongoing debates. Key themes emerging from the literature include the adaptation of existing balloon-expandable and mitral-specific devices to the complex anatomy of calcified mitral annuli, the open transatrial approach as a safer alternative to extensive surgical debridement, and advances in imaging and device design aimed at reducing left ventricular outflow tract obstruction and paravalvular leak. Persistent uncertainties remain, particularly regarding patient selection, long-term valve performance, and comparisons with conventional surgical repair or replacement. Although open transatrial implantation appears technically feasible and provides favorable hemodynamic results compared with fully percutaneous procedures, reported 30-day mortality remains high (approximately 19–27%). This reflects the advanced age, frailty, and multiple comorbidities typical of this patient group rather than procedural shortcomings. Current evidence is limited, with few comparative studies and little data on valve durability. Future work should prioritize multicenter prospective registries and well-designed comparative studies to better define the role of this emerging salvage strategy.

## 1. Introduction

Mitral annular calcification (MAC) is a degenerative process leading to circumferential or partial calcific deposition at the fibrous mitral annulus and is associated with mitral valve dysfunction, conduction disturbances, and increased operative risk for mitral valve surgery [1]. The diagnosis of MAC is on the rise, especially in the elderly population, and often coexists with multiple comorbidities such as renal failure, impaired calcium–phosphorus metabolism, and diabetes [2]. Surgical mitral valve replacement (MVR) in the presence of extensive circumferential MAC is technically demanding and historically associated with significant morbidity and mortality because of risks from debridement (atrioventricular disruption, major bleeding, circumflex coronary artery lesions) and difficulty achieving secure sewing ring fixation. Data from the Society of Thoracic Surgeons Adult Cardiac Surgery Database indicate that the presence of mitral annular calcification is associated with markedly higher perioperative risk, with operative mortality approaching 6% in MAC patients undergoing conventional mitral valve replacement, even after risk adjustment [3]. These findings are consistent with institutional surgical series, where procedures requiring annular decalcification and reconstruction have reported in-hospital mortality rates above 8% [4].

The depicted challenging scenario has prompted the off-label adoption of transcatheter heart valves (THVs) intended for aortic use [5,6], and later of dedicated mitral devices [7], implanted into the mitral valve complex either percutaneously (transseptal or transapical) or surgically via an open atrial approach/open atrial Transcatheter Mitral Valve Replacement (TMVR). Those procedures are commonly referred as “Valve-in-MAC” (ViMAC). This literature review focuses specifically on open-heart implantation of THVs for severe MAC, summarizing the current evidence regarding procedural techniques, clinical outcomes, and future directions for this evolving field.

## 2. Materials and Methods

A targeted, structured search was performed in accordance with the PRISMA (Preferred Reporting Items for Systematic Reviews and Meta-Analyses) guidelines [8]. Four major clinical databases (PubMed/MEDLINE, EMBASE, Cochrane database, Google Scholar) were searched for peer-reviewed articles, case series, and registries reporting open-access/surgical implantation of transcatheter heart valves in severe MAC or broader TMVR studies that included ViMAC cohorts. Screening of titles, abstracts, and full-text articles was conducted independently by two reviewers. Any discrepancies in study selection were addressed through group discussion until agreement was reached. Only articles written in English were eligible for inclusion.

**Figure 1:**
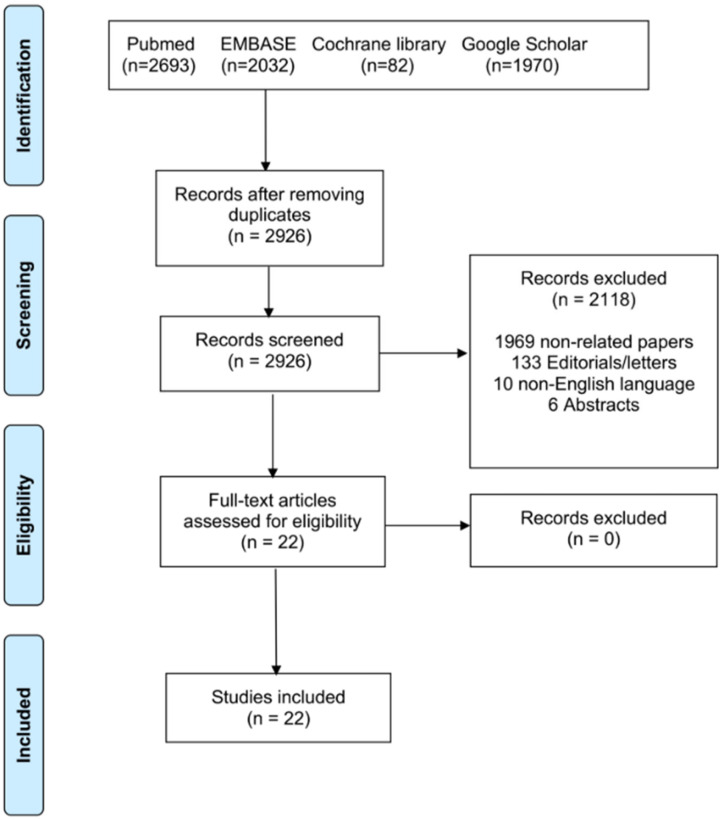
PRISMA flowchart of the included studies.

Searches covered the period from 1 January 2000 through 1 November 2025 and used combinations of keywords: “mitral annular calcification,” “transcatheter mitral valve replacement,” “valve-in-MAC,” “open atrial TMVR,” “transatrial TMVR,” “balloon-expandable valve mitral”. The search followed the PICOS framework: (1) Population: patient with severe mitral annular calcification; (2) intervention: hybrid mitral replacement/open heart THV implantation; (3) comparison: not applicable; (4) outcomes: intra-procedural success; 30-day mortality; (5) studies: all available. After deduplication and screening of titles/abstracts for relevance, full texts of potentially eligible studies were reviewed (Figure 1).

## 3. Results

This section maps how the literature and practice evolved across distinct but overlapping themes: (A) initial feasibility using balloon-expandable THVs in MAC; (B) development of the open transatrial techniques; (C) emergence of dedicated mitral TMVR devices and off-label device modification strategies (collars, anchoring skirts); (D) imaging and procedural risk mitigation (CT planning, neo-LVOT prediction, septal modification techniques); and (E) outcomes, complications and comparative effectiveness.

Early feasibility of balloon-expandable THVs in MAC: initial case reports and small series described implantation of balloon-expandable aortic THVs (e.g., Sapien family, Edwards Lifesciences, Irvine, CA, USA) into calcified mitral annuli as an alternative when surgical MVR with debridement was considered prohibitive. These early experiences established technical feasibility but also highlighted high rates of procedural complications including paravalvular leak (PVL), valve embolization, and LVOT obstruction, with substantial in-hospital mortality in early series.

Open transatrial TMVR, rationale and technique: recognizing the limits of pure percutaneous approaches in severe circumferential MAC, several centers (see Table 1) developed an open transatrial approach using cardiopulmonary bypass: via median sternotomy or less invasive access, the left atrium is opened, extensive MAC is visualized, native leaflets may be excised or preserved, and the THV is deployed under direct vision with adjuncts (felt skirts, sewing ring modifications, anchoring sutures, “extended collar” technique) to reduce PVL and improve stability. Russell et al. provided a step-by-step contemporary transatrial TMVR technique and re-ported acceptable early outcomes in high-risk patients [9]. The open approach allows controlled deployment and facilitates bail-out procedures. Of course, the major drawback of this procedure is the invasiveness of the surgical approach to the heart chambers and the use of extracorporeal circulation.

Device evolution: dedicated mitral systems and collars/anchors: while early ViMAC work was off-label use of aortic THVs, more recent years have seen the rise of dedicated mitral systems and device modifications intended for MAC anatomy [7]. Device innovation continues to target secure anchoring, sealing, and minimizing LVOT obstruction risk.

Imaging, patient selection, and procedural planning: high-resolution cardiac CT with 3-D modeling has become indispensable for TMVR in MAC: CT predicts neo-LVOT area, prosthesis-to-septum relationships, calcium distribution, and potential anchoring zones. This imaging evolution has reduced catastrophic complications and is central to patient selection criteria [10]. The CT MAC score is a systematic tool that grades the severity of mitral annular calcification (MAC) and predicts anchoring of balloon-expandable SAPIEN valves in valve-in-MAC procedures. It evaluates four anatomical features: annular calcium thickness, circumferential calcium distribution, trigone calcification, and mitral leaflet calcification [11].

**Table 1:** Clinical studies and peer-reviewed case report; NA: not available; 30 D: 30-day; 1 y: 1-year.

Authors (Year)	Country	Patients (n)	Age (Years)	STS Score or EuroSCORE II (%)	Valve Type	Successful Deployment	30-Day Mortality and Follow-Up
Carrel, 2012 [12]	Switzerland	1	81	NA	Sapien XT	100%	30 D: 0%; alive at 4 months
Astarci, 2013 [13]	Belgium	1	62	4.08%	Sapien XT	100%	No information after surgery
Ferrari, 2014 [14]	Switzerland	1	60	8%	Sapien XT	100%	No information after surgery
Murashita, 2016 [15]	USA	1	71	NA	Sapien XT	100%	Alive at discharge (day 8)
Langhammer, 2016 [16]	Switzerland	4	80/60/79/74	5/1.7/3.4/4.7	Sapien XT (3/4), Sapien3 (1/4)	100%	30 D: 0%; all alive at 4 months
Baumgarten, 2016 [17]	USA	3	89/83/85	16.8/8.6/10.7	Sapien XT (2/3), Sapien3 (1/3)	100%	All alive at discharge, no further info
Polomsky, 2017 [18]	USA	2	81/69	NA	Sapien 3	100%	1 in-hospital death, 1 discharged day 7
Alfonsi, 2017 [19]	Italy	1	76	NA	Sapien XT	100%	30 D: 0%; alive at 6 months
El Sabbagh, 2018 [20]	USA	6	81 ± 9	10.3 ± 6.0	Sapien XT (1/6), Sapien3 (5/6)	100%	30 D: 3/6 (50%)
Koehle, 2018 [21]	Germany	1	66	39%	Sapien XT	100%	30 D: 0%; alive at 1 year
Praz, 2018 [5]	USA	26	78.7 ± 7	9.4 ± 4.8	Sapien XT (2/26), Sapien3 (24/26)	100%	In-hospital: 5/26 (19%); 30 D: 7/26 (27%)
Russell, 2018 [9]	USA	8	75.6 ± 6.7	8.1 ± 3.3	Sapien 3	100%	30 D: 0%
Ahmad, 2019 [22]	Australia	3	68/67/83	NA	Sapien XT	100%	30 D: 1/3 (33%); at 18 months 2 alive
Morita, 2020 [23]	Japan	1	80	6.6	Sapien 3	100%	Alive at day 7
Albacker, 2020 [24]	Saudi Arabia	1	75	19.5	Sapien 3	100%	No information after surgery
Bagaev, 2022 [25]	Germany	6	76 ± 9	5.7 ± 1.9	Sapien 3	100%	30 D: 1/6 (16.7%); 1 y: 4 alive, 1 lost
Lamelas, 2021 [26]	USA	16	77 ± 9	7.2 ± 4.7	Sapien 3	100%	30 D: 12.5%; 1 y: 36.2%
Morita, 2021 [27]	France	1	74	NA	Sapien 3	100%	30 D: 0%; alive at 1 year
Pozzoli, 2022 [28]	Switzerland	5	70 ± 5.8	3.7 ± 2.1	Sapien 3	100%	30 D: 0%; 6 months: 0%
Hassanabad, 2022 [29]	Canada	1	79	NA	Sapien 3	100%	30 D: 0%; 6 months: alive
Smith, 2023 [30]	USA	51	73.9 ± 8	6.4 ± 4.8	Sapien XT (5/51), Sapien3 (46/51)	94.1%	30 D: 7/51 (13.7%)
Hassanabad, 2025 [31]	Canada	22	70.4 ± 9.3	3.1 ± 2.1	Sapien 3	100%	30 D: 2/22 (9%); 1 y: 18%

Outcomes, complications and comparative effectiveness: Table 1 summarizes the peer-reviewed studies and case reports describing transcatheter mitral valve-in-valve procedures performed with Sapien XT and Sapien 3 balloon-expandable prostheses between 2012 and 2024 [5,9,12,13,14,15,16,17,18,19,20,21,22,23,24,25,26,27,28,29,30,31]. The collected evidence includes both isolated case reports and small to medium-sized series, totaling more than 150 patients treated worldwide.

Procedural success was uniformly high, with successful deployment achieved in nearly all patients (≥94%, frequently 100%). This reflects the high degree of procedural control and device reliability associated with current-generation balloon-expandable valves. Despite technical success, early mortality at 30 days displayed notable heterogeneity, ranging from 0% in some low-risk cohorts to nearly 30% in early experiences or patients at very high surgical risk.

Follow-up data, though inconsistently reported, indicated satisfactory mid-term outcomes. Survival at 6 and 12 months was generally favorable, often exceeding 80%, with preserved valve function and limited structural deterioration. When comparing device generations, no major procedural differences emerged between Sapien XT and Sapien 3 valves, although later studies with the Sapien 3 suggested slightly improved procedural safety and lower periprocedural mortality, likely due to enhanced operator experience and device refinements.

## 4. Discussion

The literature reveals several ongoing controversies surrounding open-heart MVR using THVs for severe MAC. A central debate concerns whether surgical debridement of the calcified annulus should still be pursued or whether it is preferable to implant a THV while leaving the MAC largely intact. Advocates of surgical debridement argue that careful removal of calcium and annular reconstruction allow secure prosthetic fixation and reproducible long-term outcomes, particularly in younger or lower-risk patients, citing the durability of conventional mitral valve replacement when technically successful [32]. However, extensive debridement in heavily calcified or circumferential MAC carries substantial risks of annular rupture and bleeding, leading many to favor a more conservative or hybrid approach. Implanting a THV directly into the calcified annulus under direct surgical vision offers the advantages of controlled deployment, reduced paravalvular leak, and lower intraoperative risk in high-risk patients [5,9], though definitive evidence from randomized trials is still lacking.

Another widely discussed issue is the choice between off-label aortic THVs and dedicated mitral systems. Off-label use of balloon-expandable aortic valves has historically been the most common approach due to device availability and operator familiarity, yet it presents challenges of anchoring, sealing, and leaflet coaptation given the irregular mitral anatomy. Dedicated mitral prostheses such as Tendyne, Intrepid, and EVOQUE are emerging alternatives that aim to overcome these geometric and hemodynamic limitations [33]. The SUMMIT-MAC study demonstrated that the Tendyne safely and effectively improves symptoms and quality of life in high-risk patients with severe MAC despite the trial is limited by its single-arm, non-randomized design and inclusion of only centers experienced in transapical access, which may affect the generalizability of the results [34].

Further controversy centers on the risk of left ventricular outflow tract (LVOT) obstruction, one of the most feared complications of TMVR in MAC. Modern practice increasingly relies on preoperative CT-based modeling to predict the neo-LVOT area and to guide patient selection [10]. Where the predicted obstruction risk is high, several preventive strategies have been described, including pre-emptive alcohol septal ablation [35], transcatheter anterior leaflet laceration (LAMPOON) in percutaneous cases [36], or direct surgical leaflet resection when the atrium is opened. However, no uniform thresholds or standardized protocols exist.

Finally, durability and long-term performance remain major unresolved questions. Although short-term symptomatic and hemodynamic improvements are consistent [37], the long-term structural behavior of THVs implanted in the mitral position, particularly within a calcified annulus, has not been fully characterized. Authors therefore emphasize the importance of ongoing surveillance and registry data before widespread adoption in lower-risk populations.

### 4.1. Gaps in the Current Literature

The existing literature, while promising, is rudimentary and leaves many critical questions unanswered.

Lack of Comparative Data: There are no head-to-head or even robust, propensity-matched studies comparing: Hybrid THV vs. Conventional MVR (with decalcification); Hybrid THV vs. Percutaneous ViMAC; Hybrid THV vs. Medical Management.

No Long-Term Durability Data: As noted, follow-up is limited to 1–2 years in most series. The 5- and 10-year performance of these valves in the MAC-mitral position is completely unknown.

Lack of Technique Standardization: While principles are emerging, the precise technique (e.g., number of sutures, use of a skirt, type of skirt, specific valve choice) is not standardized and varies by institution [5,9,12,13,14,15,16,17,18,19,20,21,22,23,24,25,26,27,28,29,30,31].

Optimal Patient Selection: The high 30-day mortality suggests a problem of “futility”. We lack robust risk models to differentiate which patients are so frail that they will not benefit from any intervention, versus those who are robust enough to survive the hybrid operation. We acknowledge that the absence of a control group and the off-label nature of this procedure represent important limitations, highlighting both ethical considerations and the need for caution when interpreting outcomes or planning future prospective multicenter studies.

Cost-Effectiveness: This procedure uses an extremely expensive transcatheter valve in a full open-heart surgical setting. Its cost-effectiveness has not been studied.

### 4.2. Future Directions

Future research should focus on clarifying the true role of hybrid transatrial transcatheter valve implantation in severe mitral annular calcification through large, prospective multicenter registries capable of standardizing outcomes and identifying best practices. Comparative analyses using national databases are needed to benchmark its results against conventional mitral valve replacement, while long-term follow-up will be essential to assess valve durability, thrombosis risk, and reintervention rates. Continued technological development should also aim to create dedicated surgical valves optimized for sutureless implantation in heavily calcified annuli, rather than relying on devices designed for the aortic position. Ultimately, this hybrid approach should be viewed as an important salvage option for patients deemed inoperable, with its long-term future dependent on improved patient selection and durable procedural outcomes.

## 5. Conclusions

The open-heart, transatrial implantation of a transcatheter heart valve is a feasible, reproducible, and innovative hybrid solution for patients with severe MAC and prohibitive surgical risk. It successfully leverages the advantages of both surgical and transcatheter approaches: it eliminates the risk of AV groove rupture by avoiding decalcification, while simultaneously eliminating the risks of LVOT obstruction and valve embolization through direct anterior mitral leaflet resection and suture fixation. This technique delivers suboptimal hemodynamic results with minimal PVL. However, it is a major operation performed on an extremely frail and comorbid patient population, and the 30-day mortality remains high. This mortality appears to be a function of patient frailty rather than technical failure.

## Data Availability

Data compiled or analyzed throughout the study can be made available by the corresponding author upon reasonable request.

## Abbreviations

The following abbreviations are used in this manuscript:

AV	Atrioventricular
CPB	Cardiopulmonary Bypass
CT	Computed Tomography
EuroSCORE II	European System for Cardiac Operative Risk Evaluation II
LAMPOON	Laceration of the Anterior Mitral Leaflet to Prevent Outflow Obstruction
LVOT	Left Ventricular Outflow Tract
MAC	Mitral Annular Calcification
MVR	Mitral Valve Replacement
PVL	Paravalvular Leak
STS Score	Society of Thoracic Surgeons Risk Score
THV	Transcatheter Heart Valve
TMVR	Transcatheter Mitral Valve Replacement
ViMAC	Valve in MAC
